# National Biosafety Management System: A Combined Framework Approach Based on 15 Key Elements

**DOI:** 10.3389/fpubh.2021.609107

**Published:** 2021-07-23

**Authors:** Arnaud Orelle, Abdoulaye Nikiema, Arsen Zakaryan, Adilya A. Albetkova, Mark A. Rayfield, Leonard F. Peruski, Antoine Pierson, Obert Kachuwaire

**Affiliations:** ^1^Integrated Quality Laboratory Services, Lyon, France; ^2^Division of Global Health Protection, Center for Global Health, Centers for Disease Control and Prevention, Atlanta, GA, United States

**Keywords:** biosafety, safety, biosecurity, biorisk, framework, Global Health Security Agenda, capacities, system

## Abstract

The pervasive nature of infections causing major outbreaks have elevated biosafety and biosecurity as a fundamental component for resilient national laboratory systems. In response to international health security demands, the Global Health Security Agenda emphasizes biosafety as one of the prerequisites to respond effectively to infectious disease threats. However, biosafety management systems (BMS) in low-medium income countries (LMIC) remain weak due to fragmented implementation strategies. In addition, inefficiencies in implementation have been due to limited resources, inadequate technical expertise, high equipment costs, and insufficient political will. Here we propose an approach to developing a strong, self-sustaining BMS based on extensive experience in LMICs. A conceptual framework incorporating 15 key components to guide implementers, national laboratory leaders, global health security experts in building a BMS is presented. This conceptual framework provides a holistic and logical approach to the development of a BMS with all critical elements. It includes a flexible planning matrix with timelines easily adaptable to different country contexts as examples, as well as resources that are critical for developing sustainable technical expertise.

## Introduction

Before the recognition of the importance of biosafety at the World Health Organization's 58th World Health Assembly in 2005, biosafety has received increased attention in global health capacity building projects. Major international efforts have sought to advance a world safe and secure from infectious disease threats (1–4), especially as shown through the Global Health Security Agenda (GHSA), an international partnership of nearly 70 countries and major international organizations (such as WHO, OIE, FAO) engaged against infectious diseases (1). This GHSA encourages countries around the world to make new concrete commitments and transform global health security in support of the International Health Regulations (IHR) (5). Composed of 11 “action packages,” including biosafety and biosecurity, GHSA aims to reduce the ability of dangerous pathogens spreading rapidly within and across borders (2).

In the biosafety-biosecurity action package of the GHSA, the overarching goals are the: “Implementation of a comprehensive, sustainable and legally embedded national oversight program for biosafety and biosecurity, including the safe and secure use, storage, disposal, and containment of pathogens found in laboratories and a minimal number of holdings across the country, including research, diagnostic and biotechnology facilities. A cadre of biological risk management experts possesses the skillset to train others within their respective institutions. Strengthened, sustainable biological risk management best practices are in place using common educational materials. Rapid and culture-free diagnostics are promoted as a facet of biological risk management. The transport of infectious substances will also be taken into account” (6).

Despite these concrete targets, the biosafety-biosecurity action package is silent on concrete actions. The design and implementation of the requisite laboratory biosafety and biosecurity programs is inconsistent globally, especially in low- and middle-income countries (LMIC), with major gaps influenced by a variety of factors that include differences in national and local infrastructures, available funding and priorities, regulatory frameworks, and accessibility to expertise, training and equipment resources (3).

Many institutions, organizations and countries have worked on the development of biosafety management systems (BMS). BMS is based on a management system approach, which enables an organization to effectively identify, assess, control, and evaluate the biosafety and biosecurity risks inherent in its activity (7). However, they typically have focused only on few key elements of a national BMS, usually due to various constraints, such as budgetary limitations, time, or simply a limited understanding of what is a BMS (4–6, 8). Lessons from our extensive work in Burkina Faso (development of a national biosafety guideline, development of a biosafety and biosecurity assessment tool, conducting biosafety and biosecurity assessments, development of national biosafety regulations, and training of national assessors); other collaborations in Armenia, Burundi, Cameron, Guinea, Ghana, Georgia, Laos, Mauritania (organization of biosafety support, trainings and or assessments); Liberia and India (biosafety training, development of a national biosafety guideline, and specific support); Morocco (development of an laboratory infrastructure guideline); Sudan, Myanmar and Ethiopia (BSL3 assessments and/or trainings) (all unpublished), as well as observed gaps in various project countries which include: inadequate biosafety policies, lack of biosafety cabinet certification programs, lack of pre-service laboratory biosafety training curricula, absence of biosafety training packages and cascade training, and lack of IATA certified international shipment of infectious material capacities, have led us to a comprehensive and systems-based approach for a national BMS that can be sustainable and country-owned. Borrowing from our extensive global experience, we propose a conceptual framework outlining key steps to fulfill the biosafety expectations under the GHSA. We provide details to effectively strengthen biosafety and biosecurity capacities, through the development of sustainable biosafety management system (BMS) at country level.

## Development of the System and Supporting Framework

### Fundamental Considerations

There is of course no one-size-fits-all strategy to a biosafety management system; however, we identified common, key elements critical to establish a BMS. These 15 elements will fully capacitate a country in biosafety and biosecurity:

1. Creation of a National Biosafety Committee (NBC) and nomination of a National Biosafety Focal Person (NBFP) and a deputy NBFP
2. Development of biosafety and biosecurity national policies
3. Monitoring and evaluation of the implementation of the biosafety management framework
4. Organization of a biosafety and biosecurity training of trainers (for the core team of implementers)
5. Adaptation and development of the biosafety training package (for central and decentralized levels)
6. Identification, adaptation or development of a biosafety and safety laboratory assessment tool (BSS-LAT)
7. Conduct a national biosafety assessment using the developed BSS-LAT
8. Training of national BSS officers on assessment processes using the BSS-LAT
9. Development of a national biosafety and biosecurity guideline
10. Implementation of cascade trainings on biosafety and biosecurity at subnational levels
11. Adaptation and development of comprehensive national regulations on biosafety and biosecurity
12. Adaption, development, harmonization of national Standard Operating Procedures (SOPs) for biosafety and biosecurity
13. Reviewing and ensuring appropriate IATA training and certification at national level
14. Development or strengthening of (national) capacity of biomedical engineers to maintain biosafety equipment
15. Development and implementation of biosafety curricula for initial and recurrent trainings.

The key elements of the BMS framework that we propose are presented in Table 1. A number of them are interrelated; all we have found to be essential in establishing a sustainable and country-owned BMS. In addition to these 15 elements, there are three additional or supplemental elements that could be included in the BMS process but are less critical:

(a) Organization of a workshop on pathogen risk group classification,
(b) Development of a laboratory infrastructure guidelines, and
(c) Organization of a workshop on biobanking, including regulation and data protection.

**Table 1:** Proposed combined framework approach based on 15 key elements, and detailed sub-activities.

	**Key element - main activities**	**#**	**Sub-activities (suggested, not exhaustive)**	**Proposed timeline**
1	Create a National Biosafety Committee and Nominate a Country Biosafety Focal Person	1.1	Identify the key national biosafety, safety and biosecurity stakeholders (MoH, MoA, MoE, Institutions, Partners).	Month 0
		1.2	If not existing, establish a national laboratory governance and oversight system that will take care of Biosafety and Biosecurity implementation (usually part of the laboratory governance)	Month +1
		1.3	Constitute a national laboratory biosafety/biosecurity committee (the committee can include committee, department, agencies). In some countries, it may be recommended to mandate existing government agency to administer and enforce BSS (Example of Agencies dealing with only GMO)	Month +2
		1.4	Develop TOR for the committee, appoint and capacitate a national biosafety/biosecurity committee, actors to be identified from cross-cutting ministries. The ToR should include clear goals, vision and objectives.	Month +3
		1.5	Nominate a Biosafety Country Lead person (“Biosafety Focal Person”), and identify a deputy (recommended to have both MoH and MoA representatives)	Month +4
		1.6	Develop a generic job description (and ToRs) for Biosafety Focal Person and the deputy	Month +5
		1.7	Decide for a core team of biosafety implementers (TBI) (5–12 persons)	Month +6
		1.8	Identify/look for funds allowing sustainability of the committee	Month +6
		1.9	Develop a plan of action with clear sub-activities, timeline, budget and responsibilities	Month +7
		1.10	Decide for a core team of biosafety implementers (TBI) (5–12 persons), recommended to have both MoH and MoA representatives	Month +7
		1.11	Organize regular meetings (minimum bi-annually)	/
2	Develop Biosafety and Biosecurity National Policies	2.1	Develop National Biosafety and Biosecurity Policies	Month +5
		2.2	Share National Biosafety and Biosecurity Policies nationally	Month +6
3	M&E – Monitoring and Evaluation: Definition of indicators for Performance of the National Biosafety Committee	3.1	Validation of the plan of action	Month +7
		3.2	Identification of performance indicators for each activity	Month +8
		3.3	Evaluation of the performance of the indicator on regular basis (at least every year)	Month +12
4	Organize a Biosafety and Biosecurity Training of Trainers (for TBI)	4.1	Decide for the topics to be included	Month +3
		3.2	Decide for participants (core team of biosafety implementers at minimum)	Month +5
		3.3	Budgeting for the training	Month +5
		3.4	Organization of the Training	Month +6
5	Adapt/Develop the biosafety training package (for Central and Regional level)	5.1	Assess the training (ToT) received	Month +7
		5.2	Modify the training materials for Central and Regional level	Month +8
		5.3	Validate the training materials (finalized training package)	Month +10
		5.4	Develop a training manual for trainers	Month +12
6	Identification, Adaptation or Development of a Biosafety Laboratory Assessment Tool (BSS-LAT)	6.1	Identify existing Biosafety Laboratory Assessment tools	Month +8
		6.2	Assess the relevancy of the existing tools (Review)	Month +9
		6.3	Workshop to create a country Biosafety Laboratory Assessment tool (use an existing one/update an existing one/develop one) based on the needs	Month +10
		6.4	Validation of the BSS-LAT	Month +11
7	Biosafety Assessment (with mentor) using the developed BSS-LAT	7.1	Assess the main laboratories of the country (central and regional) using the BSS-LAT (if possible with mentor)	Month +13
		7.2	Generate a report of assessments (individuals, for each lab assessed), and generic, highlighting the main deficiencies	Month +15
		7.3	If required, modify the BSS-LAT	Month +16
8	Train national BSS Officers on assessment process (Mentored assessment)	8.1	Identify the persons to be trained as BSS officers (Recommended TBI)	Month +18
		8.2	Train and mentor national BSS officers on BSS audit and use on the BSS-LAT	Month +20
		8.3	Develop national Biological Safety Officer Certification program	Month +22
		8.4	Recognize the National BSS officers	Month +24
9	Develop a National (bio)safety and (bio)security guideline	9.1	Decide for the scope of action (applicable for clinical, public health, human, vet, food/water/environment laboratories)	Month +16
		9.2	Decide for a template/model to be used	Month +18
		9.3	Organize a Workshop to revise the guideline	Month +22
		9.4	Validate the guideline	Month +28
		9.5	Disseminate the guideline	Month +34
10	Implement the cascade training on Biosafety and Biosecurity at Regional level	10.1	Organize a biosafety and biosecurity training in each Region	Month +13
		10.2	Assess the training received at regional level	Month +14
		10.3	If relevant, decide for a core team of Regional Biorisk implementers (TRBI)	Month +16
		10.4	If relevant, modify the training materials for district level	Month +18
		10.5	Develop a training manual (district level) for trainers from regional level	Month +20
		10.6	Validate the training materials for district level, plan and organize cascade training	Month +24
11	Adapt/Develop comprehensive national regulations on Biosafety and Biosecurity	11.1	Review the existing regulations (laws/decrees) about safety/biosafety (recommend to be developed or refined only after biosafety management system is already partially implemented, to better refine national strategy and associated regulations)	Month +15
		11.2	Decide for the approach to be followed for the development of regulations (revision of act/law; new act; new decree)	Month +16
		11.3	Decide for the topics to be covered by the regulations	Month +17
		11.4	Organize a workshop to draft the regulations	Month +19
		11.5	Validate the biosafety regulations	Month +20
12	Adapt/Develop/Harmonize Biosafety and biosecurity National SOPs	12.1	Decide for the main national procedures to be developed for Biosafety and Biosecurity (topics)	Month +18
		12.2	Preparation of the national SOPs	Month +19
		12.3	Review of the national SOPs	Month +20
		12.4	Validation of the national SOPs	Month +22
		12.5	Dissemination of national SOPs	Month +24
13	IATA Training	13.1	Make an inventory of the staff at country level (and more importantly at central level) with IATA certification	Month +12
		13.2	Review the IATA training needs for the Department/Institution (ToT or Simple training)	Month +15
		13.3	Identify IATA training possibilities (could be through partner or WHO)	Month +18
		13.4	Organize IATA training for the identified staff	Month +24
14	Develop/revive local (national) capacity of biomedical engineers to maintain biosafety equipment (most important is BSC certification)	14.1	Perform a review/identify national capacities (people trained/to train)	Month +10
		14.2	If relevant, perform a root-cause analysis on the reason why the previous implementation was not successful	Month +12
		14.3	Perform an inventory (estimation of number and location) of BSC in the country	Month +18
		14.4	Estimate the BSC certification needs in the country	Month +20
		14.5	Estimate the costs of developing or reviving the national capacities (may include re-training of staff/ buying additional certification equipment/ calibration of some equipment)	Month +23
		14.6	Estimate the annual costs for BSC certification in the country	Month +24
		14.7	Develop national capacities for BSC certification (include training of staff/ buying certification equipment/ calibration of some equipment), if any existed, revive	/
15	Update of the curricula to implement or update biosafety initial training	15.1	Identify/determine the existence/gaps of biorisk curricula in educational institutions	Month +24
		15.2	Develop and officialize a national harmonized curriculum (through University/ Relevant Ministry, such as Ministry of Education)	Month +30
16	Organize a Workshop on pathogen risk group classification (Human, Animal and Environmental pathogens)	16.1	Organization of a workshop on pathogen risk group classification for human, animal and environmental pathogens	Month +18
		16.2	Issue an official classification of pathogen by risk group (for human, animal and environment)	Month +24
17	Development of a Laboratory infrastructure guidelines	17.1	Make a review of possible documents already existing in other countries	/
		17.2	Decide for the best approach (development from scratch, adaptation of other documents)	/
		17.3	Organize a workshop to develop a guideline with criteria and specification on laboratory infrastructures	/
		17.4	Validation of the guideline	/
18	If relevant, Biobank workshop [Regulations + Data protection (MTA)]	18.1	Identification of Laboratory/ies to serve as a Biobank	/
		18.2	Assess the security of the biobank laboratory	/
		18.3	Determine the gaps and ensure the biobank lab is up to date	/
		18.4	Determine the necessary equipment, logistics and structural renovations	/
		18.5	Train personnel to manage the biobank	/

The timeline proposed is intentionally aggressive in order to both elevate the importance of BMS in the national strategy and to prioritize the identification of gaps. Key contextual factors that should be considered by countries following this framework and timeline include, the size of the country, budget available, time dedicated by persons involved, and ease of the collaboration between governmental institutions and partners.

### Development of Sustainability and Country Ownership

Implementation requires mentorship and guidance through the National Biosafety Committee and an implementation team composed of national or international specialist(s). In our experience, external technical assistance through best practices, examples, and objective feedback is beneficial. Public or private institutions, including professional biosafety associations can provide this expertise (9–11). Even with external technical assistance, the country should continue to maintain ownership of the implementation process, including leadership of the process, typically through the National Biosafety Focal Person (NBFP) and/or a National Biosafety Committee (NBC) (per our framework). Such leadership is an important aspect of national capacities (7), and is critical for implementation. Absence of leadership makes implementing a comprehensive national BMS all but impossible.

### Development of Training

A major gap in developing and implementing a national BMS is the paucity of local knowledge and skills. This gap can be addressed through training of local experts who will become the champions and leaders of the BMS. Numerous training packages exist and can be delivered through various mechanisms – in-class training or online, either free or for a fee (12). Additionally, many reputable organizations provide biosafety training. Based on our experience, we recommend countries ensure training is performed according to local needs and requirements, that it includes identified topics of interest, and delivered according to the modalities defined by country policies and customs. These should include at a minimum simulation exercises that explore theoretical and practical aspects, opportunities for facilitated discussion and exchange of information, and include training materials in a format that allows updating and adaptation (such Microsoft Office, Google Docs). As with other activities mentioned here, support from external partners can be beneficial, provided leadership and ownership from the country is maintained.

A growing number of remote or virtual training solutions have become available in recent years and can even be used as curricula base (13). However, these tools should be thoroughly reviewed and preference given to materials of quality and adaptable to the local context. Public platforms such as YouTube? have also became a vast resource of training materials, here again, with a broad range of quality, but amendable to inclusion in training plans.

### Locating Resources and Tools

Additional supporting resources (including training materials) and tools are widely available such as those offered by the Centers for Disease Control and Prevention (14), the World Health Organization (15), the U.S. National Institutes of Health (9), the Association of Public Health Laboratories (10), the American Biosafety Association (11), the International Federation of Biosafety Associations (16) and even universities [examples include the University of Tennessee (17), Yale University (18)]. Private agencies have also developed numerous biosafety resources. The difficulty is not in finding resources, but rather in identifying high-quality resources that can be adapted to the specific country context.

### Dedicated Financial Resources Are Essential

A dedicated budget is vital to ensure that the proposed activities can be executed. Funds are critical to support training, infrastructure requirements including procurement of safety, information technology equipment, facility renovations, and implementation and monitoring activities. These financial resources should ideally be identified and driven from various channels, including government/ministry funding, vertical programs and partners. This approach to funding will help ensure collaboration of efforts and partners and reduce duplication or unnecessary activities that often are counterproductive to the end goals.

## Toward a Sustainable System

We strongly believe that taking into consideration all 15 key elements from our framework is critical. Additionally, these actions should be developed in an integrated manner, ensuring the establishment of a sustainable system that can be country-owned. This BMS can be independent on individual expertise or donor projects, as it takes a long-term perspective and enhances national capacities and competencies on biosafety and associated skills.

Our team has been involved in implementing a number of these key elements of a biosafety management system through various international projects (unpublished). Lessons learnt through our experiences strongly suggest an integrated approach that considers all aspects of a BMS is optimal. To our knowledge this type of approach has not been implemented nor described previously. We offer this perspective and guidance to assist future projects, implementers and stakeholders to view the BMS in its totality using the critical elements that we have defined. In this way, it will ensure all the interrelated components of a national BMS are addressed and implemented in sustainable fashion to achieve the goals of the GHSA.

## Data Availability Statement

The original contributions presented in the study are included in the article/supplementary material, further inquiries can be directed to the corresponding author/s.

## Author Contributions

The original concept of the manuscript was developed by AO, OK, and AP, who also drafted the manuscript. AN, AZ, AA, and LP contributed to the development of the approach presented in the manuscript and provided comments and revisions to the draft manuscript. All authors contributed to the article and approved the submitted version.

## Conflict of Interest

The authors declare that the research was conducted in the absence of any commercial or financial relationships that could be construed as a potential conflict of interest.

## Publisher's Note

All claims expressed in this article are solely those of the authors and do not necessarily represent those of their affiliated organizations, or those of the publisher, the editors and the reviewers. Any product that may be evaluated in this article, or claim that may be made by its manufacturer, is not guaranteed or endorsed by the publisher.
